# Synergistic Approach of Ultrafast Spectroscopy and Molecular Simulations in the Characterization of Intramolecular Charge Transfer in Push-Pull Molecules

**DOI:** 10.3390/molecules25020430

**Published:** 2020-01-20

**Authors:** Barbara Patrizi, Concetta Cozza, Adriana Pietropaolo, Paolo Foggi, Mario Siciliani de Cumis

**Affiliations:** 1 National Institute of Optics-National Research Council (INO-CNR), Via Madonna del Piano 10, 50019 Sesto Fiorentino, Italy; patrizi@lens.unifi.it (B.P.); foggi@lens.unifi.it (P.F.); 2 European Laboratory for Non-Linear Spectroscopy (LENS),Via Nello Carrara 1, 50019 Sesto Fiorentino, Italy; 3 Dipartimento di Scienze della Salute, Università di Catanzaro, Viale Europa, 88100 Catanzaro, Italy; concetta.cozza@studenti.unicz.it (C.C.); apietropaolo@unicz.it (A.P.); 4 Dipartimento di Chimica, Biologia e Biotecnologie, Università di Perugia, Via Elce di Sotto 8, 06123 Perugia, Italy; 5 Italian Space Agency, Contrada Terlecchia snc, 75100 Matera, Italy

**Keywords:** ultrafast spectroscopy, push-pull molecules, DFT, TD-DFT, transient absorption spectroscopy, ICT, two-dimensional electronic spectroscopy, machine learning, molecular simulations

## Abstract

The comprehensive characterization of Intramolecular Charge Transfer (ICT) stemming in push-pull molecules with a delocalized π-system of electrons is noteworthy for a bespoke design of organic materials, spanning widespread applications from photovoltaics to nanomedicine imaging devices. Photo-induced ICT is characterized by structural reorganizations, which allows the molecule to adapt to the new electronic density distribution. Herein, we discuss recent photophysical advances combined with recent progresses in the computational chemistry of photoactive molecular ensembles. We focus the discussion on femtosecond Transient Absorption Spectroscopy (TAS) enabling us to follow the transition from a Locally Excited (LE) state to the ICT and to understand how the environment polarity influences radiative and non-radiative decay mechanisms. In many cases, the charge transfer transition is accompanied by structural rearrangements, such as the twisting or molecule planarization. The possibility of an accurate prediction of the charge-transfer occurring in complex molecules and molecular materials represents an enormous advantage in guiding new molecular and materials design. We briefly report on recent advances in ultrafast multidimensional spectroscopy, in particular, Two-Dimensional Electronic Spectroscopy (2DES), in unraveling the ICT nature of push-pull molecular systems. A theoretical description at the atomistic level of photo-induced molecular transitions can predict with reasonable accuracy the properties of photoactive molecules. In this framework, the review includes a discussion on the advances from simulation and modeling, which have provided, over the years, significant information on photoexcitation, emission, charge-transport, and decay pathways. Density Functional Theory (DFT) coupled with the Time-Dependent (TD) framework can describe electronic properties and dynamics for a limited system size. More recently, Machine Learning (ML) or deep learning approaches, as well as free-energy simulations containing excited state potentials, can speed up the calculations with transferable accuracy to more complex molecules with extended system size. A perspective on combining ultrafast spectroscopy with molecular simulations is foreseen for optimizing the design of photoactive compounds with tunable properties.

## 1. Introduction

In the last decades, organic molecules with a delocalized π-system of electrons have been extensively investigated because they are attractive targets for application in different fields of advanced functional materials. Push-pull organic molecules are characterized by an electron-withdrawing substituent, the acceptor (A), and an electron-donating substituent, the donor (D), linked by π conjugated bridges. This kind of structures makes the π bond very polarized so that the rotational barrier for a push-pull molecule becomes lower with respect to ordinary molecules. This unique characteristic makes these molecules interesting candidates as logic gates [1], sensors [2,3], photoswitches for potential theranostic applications [4,5], photovoltaic devices [6,7,8,9], field-effect transistors [10], as well as light-emitting diodes [11].

Recent papers have focused on the study of the π-conjugated linkers, which can modulate the HOMO and LUMO energy levels, facilitating light-harvesting of these molecular complexes [12,13,14]. On the other hand, the photochemical properties, i.e. the fluorescence quantum yield, the fluorescence lifetime and the band gap, are strongly dependent on the nature of the D–A Intramolecular Charge Transfer (ICT) [7,8,15]. The ICT states differ from the parent ground states for the electronic structure and often for the molecular geometry. 

**Figure 1:**
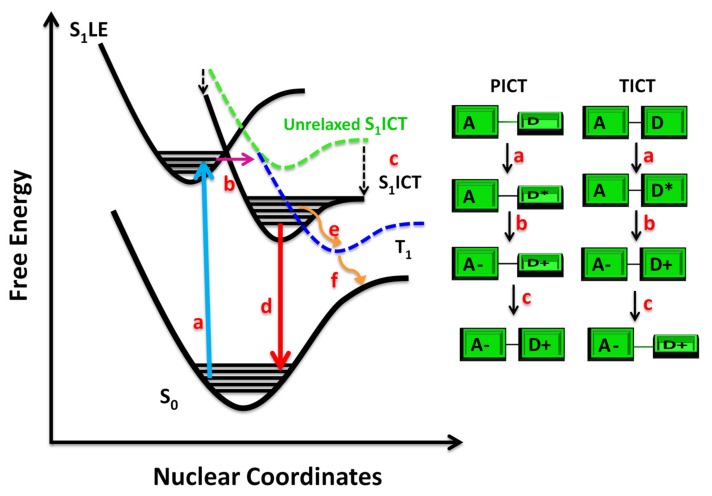
Scheme depicting the evolution of the Intramolecular Charge Transfer (ICT) in push-pull molecules. S_0_ is represented by the black curve located at the bottom. (**a**) Vertical excitation from S_0_ to a Locally-Excited S1 state (S_1_LE). (**b**) ICT from S_1_LE to an unrelaxed S_1_ICT state. (**c**) The unrelaxed S_1_ICT state can be stabilized by geometrical rearrangements (PICT or TICT) and by solvation. (**d**) Decay of S_1_ICT state through fluorescence emission. (**e**) Inter System Crossing (ISC) from S_1_ICT state to a Triplet state (T_1_). (**f**) Relaxation of T_1_ state to S_0._

Photo-induced Charge Transfer (CT) emissive states, evolving from Locally Excited (LE) states, are characterized by a large variation of the electric dipole moment between ground (S_0_) and first excited state (S_1_) which is responsible for the solvatochromic effects. Figure 1 reports a scheme depicting the evolution from S_0_ to a S_1_LE state evolving in a solvent stabilized CT state giving rise to a lower energy S_1_ emissive state.

The CT state emission is often accompanied by large Stokes shift as a consequence of solvation dynamics, especially in polar solvents [6,8,16].

Time-dependent Stokes shift measurements provide information on dielectric relaxation dynamics, which takes place from the electrostatic interactions between the molecule dipole with the surrounding polar solvent molecules and gives rise to a frequency dependence of the dielectric relaxation.

The photo-induced ICT state can be accompanied by structural reorganization like the rotation of chemical groups in the so-called Twisted Intramolecular Charge Transfer (TICT)[17,18] or by the planarization of molecule structure, i.e., the Planar Intramolecular Charge Transfer (PICT) [7,8,9] (see Figure 1). The structural rearrangements are influenced by the solvent polarity which plays an essential role in stabilizing the ICT state through the reorganization of the molecular solvation shell. From an electronic point of view, the TICT involves a large charge separation because the mesomeric interaction between D and A is blocked owing to their reciprocal twisting, while the PICT envisages a quinoidal structure with partial positive charges on the D and A groups. The extent of charge separation depends on the torsional angles [17,19] and influences the fluorescence quantum yields of these molecular systems. For many applications, a high fluorescence quantum yield and a large Stokes shift are required at the same time. The latter can be achieved by employing compounds which show a twisted geometry in the excited state however, it must be taken into account that large twisting decreases the fluorescence quantum yield as the emission from the twisted state is forbidden. On the other hand, some push-pull molecules undergoing PICT show a twisted geometry in the ground states and planar in the excited states, giving rise to very large Stokes shift and high fluorescence quantum yields at the same time [7,9,20]. In the first part of this review, we report on femtosecond Transient Absorption Spectroscopy (TAS) as a powerful tool to study the photophysics of push-pull molecules, the nature of the ICT state and to understand how the environment polarity influences radiative and non-radiative decay mechanisms in the time scale ranging from femtoseconds to nanoseconds. The above-mentioned section will be followed by a brief section on the recent advances of multidimensional ultrafast spectroscopy, in particular Two-Dimensional Electronic Spectroscopy (2 DES), and its application in unraveling ICT nature in push-pull molecular systems. In the second part of the review, we discuss the simulation and modeling approaches, which have provided, over the years, significant information on photo-excitation, emission, charge-transport and deactivation pathways of push-pull molecules. 

Density Functional Theory (DFT) coupled with the Time-Dependent (TD) framework has been successfully applied to describe electronic properties and dynamics of molecules but only for a limited system size. Recently, Machine Learning (ML) or deep learning approaches as well as free-energy simulations containing excited state potentials, have demonstrated that is possible to speed up the calculations with transferable accuracy to more complex molecules, with extended system size. In this framework, we report an outlook on ML-based methods and large-scale simulations to predict and deeply understand the photophysics of complex push-pull molecular systems.

## 2. Basic principles of Transient Absorption Spectroscopy (TAS) and Its Application to Study Push-Pull Molecules

**Figure 2:**
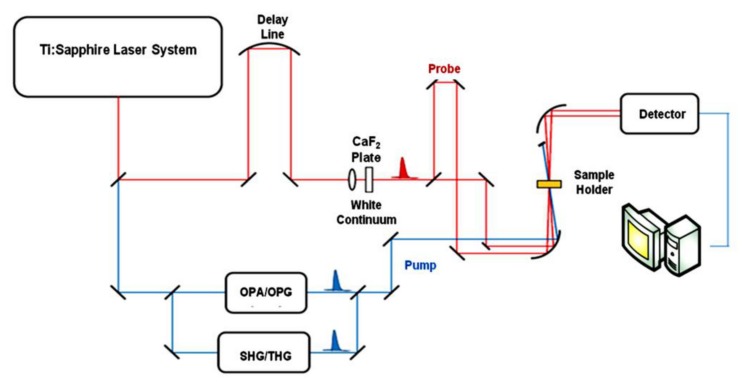
Scheme of a typical Transient Absorption Spectroscopy (TAS) set-up.

Nowadays, mode-locked Ti:sapphire lasers can routinely provide highly stable pulses, even as short as about 4 fs. The resulting fundamental 800 nm laser mode can be efficiently tuned in the UV and visible spectral region in order to provide suitable pump wavelengths [7,21,22,23]. The wide tunability is accomplished by means of OPA (Optical Parametric Amplification), OPG (Optical Parametric Generation) or by Second Harmonic (SHG) and Third Harmonic (THG) Generation. In Figure 2 the scheme of a typical TAS set-up is presented. The OPA is pumped with the amplified pulses at the fundamental or doubled frequency from the Ti:sapphire laser. Inside the OPA, these pulses are first down-converted into the near-infrared and then up-converted into the visible or UV spectral range with relatively high pulse energies by using non-linear mixing processes such as frequency-doubling, sum-frequency generation, and difference-frequency generation in suitable non-linear crystals [24,25,26,27].

In order to detect the pump-induced absorbance changes a part of the amplified 800 nm light is focused on a sapphire or calcium fluoride plate for generating a white-light continuum (400–1100 nm) which is used as a broadband probe. The probe is then focused on the sample spatially overlapped with the pump and both are collimated toward the spectrograph.

In a typical pump-probe experiment a fraction of the molecules is promoted to an electronically excited state by means of a suitable pump pulse, while the probe pulse is sent through the sample with a delay τ with respect to the pump pulse. In this way the difference absorption spectrum (the absorption spectrum of the excited sample minus the absorption spectrum of the sample in the ground state, i.e., Δ*A* spectrum) is obtained. By varying the time delay between the pump and probe and recording the Δ*A* spectrum at each time delay it is possible to obtain information on the dynamic processes occurring in the molecule under study in a time scale usually ranging from femtoseconds to nanoseconds.

The Δ*A* spectrum is characterized by common features due to various processes: Ground State Bleaching (GSB), Stimulate Emission (SE), Excited State Absorption (ESA) and Product Absorption (PA).

GSB is a *negative* signal in the Δ*A* spectrum which is observed in the wavelength region of ground state absorption. This signal is due to the fraction of the molecules which has been promoted to the excited state through the pump pulse, i.e., it corresponds to the ground state population depletion. 

In the case of push-pull molecules the GSB signal corresponds to the CT band bleaching [7,9,28] and it appears instantaneously upon photo-excitation. Also, SE is a negative signal in the Δ*A* spectrum. In the SE process, a photon from the probe pulse induces emission of another photon from the excited molecule, which returns to the ground state. Stimulated emission will occur only for optically allowed transitions and corresponds to the fluorescence spectrum of the excited molecule. SE is Stokes-shifted with respect to the ground-state bleach, even if sometimes it spectrally overlaps with ground-state bleach signal. Frequently in push-pull molecular systems this signal undergoes to a dynamical Stokes shift over the time (in a sub- and picoseconds time scale) as a consequence of solvation dynamics involving the chromophore in the S_1_ CT state and the solvent molecules [7,19,28,29,30]. Very often the solvation can be accompanied by conformational relaxation such as PICT or TICT as discussed in the introduction. 

ESA is a *positive* signal in the Δ*A* spectrum. This signal is due to optically allowed transitions from excited populated states of a molecule to higher excited states in wavelength regions generally different from ground state absorption.

PA of transient or a long-lived molecular state, such as triplet states, charge-separated states, and isomerized states appears as a *positive* signal in the Δ*A* spectrum. 

TAS has been used as a tool in studying different class of push-pull molecules, especially for clarifying the ICT nature occurring after photo-excitation. In the next section, we comment upon recent literature dealing with ICT state in push-pull molecules studied with UV-visible TAS. 

### Study of the Photophysical Properties and Excited State Dynamics of Push-Pull Molecules by UV-vis TAS from the Recent Literature

The structural simplest dyes undergoing ICT are characterized by single electron-donating and electron-accepting groups connected by a π-conjugated linker. Multi-branched and more extended push-pull molecular systems give rise to peculiar electronic properties compared to the linear systems such as fast spectral response, efficient movement of charge carriers and exciton diffusion through the entire backbone.

A lot of multi-branched chromophores have been synthesized with varying donor-π-acceptor configurations, as well as different π-bridging units, and different donor-acceptor strengths in order to achieve different structure-function relationships [31,32].

Recently theoretical and experimental studies have contributed to the description of the structure-function relationships in push-pull systems with respect to solvent effects on the ICT dynamics [7,29,31,33].

In this frame, by employing femtoseconds TAS, it is possible to follow the gradual transition from the LE state to the ICT state and to understand how solvent polarity influences excited states dynamics and its deactivation pathways. 

**Figure 3:**
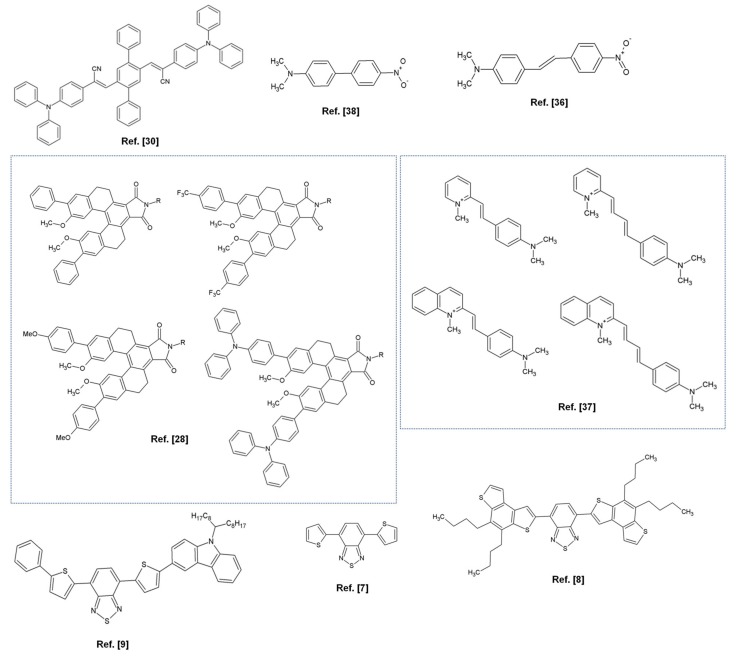
Molecular structures of push-pull systems analyzed in Section 2 with the relative references numbers. In the molecular structures of [28] R—dodecyl.

Song et al. reported the relaxation dynamics of the excited states of a cyano-substituted oligo-α-phenylenevinylene-1,4-bis(*R*-cyano-4-diphenylaminostyryl)-2,5-diphenylbenzene (CNDPASDB) studied by UV-vis TAS [30]. The molecular structure of the above-mentioned molecule and those of the other push-pull systems here reviewed are depicted in Figure 3.

Song et al. found that in low-polarity solvents fluorescence emission of CNDPASDB was mainly due to the emission from a LE state, whereas in high-polarity solvents non-radiative decay from the CT state dominates. DFT/TD-DFT analysis also confirmed an efficient intercrossing of LE and CT states with increasing solvent polarity. These results provided information for understanding the relationship between solvent polarity and the Hybridized Local excitation and Charge Transfer (HLCT) [30]. 

The ICT state and its decay mechanisms in two push-pull benzothiadiazole based compounds has been recently investigated in polar and non-polar solvents by employing TAS and TD-DFT calculations [7,8]. UV-vis TAS of 4,7-dithien-2-yl[2,1,3]benzothiadiazole (DTB) [7] and 4,7-dithien-2-yl[2,1,3]benzothiadiazole (F500) [8] (see Figure 3) revealed the instantaneous formation of a LE S_1_ state (accompanied by a big change in the dipole moment with respect to S_0_), which undergoes a PICT in a time scale ranging from hundreds of femtoseconds to few picoseconds depending on solvent polarity. The strong dipole-dipole interactions with the polarized solvent molecules stabilize the S_1_ CT state that decays principally through fluorescence emission. The fluorescence lifetimes were substantially longer in polar solvents for both the molecules [7,8] and the fluorescence quantum yield were higher for F500 in polar solvents [8]. The authors concluded that the radiative relaxation time increases when molecular planarization of the S_1_ CT state took place and that PICT is favored in polar solvents where local dipole-dipole interactions support the structural stabilization of a planar CT emissive state. 

Similar findings have been found by Scarongella and coworkers which reported a TAS study on CDTBT ((4-(5-(*N*-(9-heptadecanyl)carbazol-2-yl)thiophen-2-yl)-7-(5-phenylthiophen-2-yl)benzo[2,1,3]thiadiazole)) [9].

The authors found that the non-relaxed S_1_ state was populated *through* ultrafast (<200 fs) internal conversion. TA spectra highlighted the presence of relaxation mechanisms leading to an increase of the ICT character in polar solvents. Similarly to what observed for DTB and F500 also CDTBT S_1_ state decay mechanisms are dominated by a solvation driven and stabilized PICT responsible for a substantial dipole increasing in the S_1_ state [9]. 

Zhu et al. [28] reported about the excited-state ICT characteristics of four tetrahydro[5]helicene-based imide (THHBI) derivatives with various electron-donating groups (Figure 3) in different polarity solvents using steady-state spectroscopy and TAS. The authors found small bathochromic-shift of the absorption spectra but large red shift of the emission spectra for all dyes with increasing solvent polarity and larger dipole moments of the excited states compared to ground states. TA spectra recorded as a function of electron-donating substitutes and solvent polarity highlighted that the molecules with stronger donors (THHBI-triphenylamine (PhNPh_2_)) in more polar solvent underwent faster excited-state ICT relaxation, leading to the formation of solvent-stabilized ICT state which decays by fluorescence emission to the ground state (Figure 1). The dyes with relatively weaker donors showed instead a weaker dependence on solvent polarity, and an Inter System Crossing (ISC) from ICT state to the triplet state [28] (see Figure 1).

From the calculated Potential Energy Surface (PES) of the ground and excited states, decay times and dynamics can be predicted through ab-initio molecular dynamics [34,35], where the molecular coordinates evolve during time in a given electronic state potential energy surface.

In these regards, the excited state dynamics of a structurally flexible *push–pull* stilbene, namely, *trans*-4-(N,N-dimethylamino)-4′-nitrostilbene (DMANS), has been reported varying the solvent polarity by Sing et al. [36]. After photo-excitation of the planar DMANS molecule to the S_1_ state, it undergoes cis–trans isomerization through the twisting of the double bond. The TICT was the dominant relaxation processes in the S_1_ state of DMANS, in polar solvents.

Different D–π–A cationic systems with a methyl pyridinium or quinolinium as the electron-deficient group, a dimethyl amino as the electron-donor group, and an ethylene or butadiene group as the spacer have been investigated in a joint spectroscopic and TD-DFT computational study by Carlotti and colleagues [37]. The authors found that fluorescence efficiency decreased with solvent polarity and that the excited-state optimized geometries were planar in low-polarity media and twisted in high-polarity media. Femtoseconds TAS disclosed the occurrence of a fast photo-induced ICT followed by a fast vibrational relaxation which takes place on a time scale of several hundreds of femtoseconds from the upper vibrational states of the singlet ICT state (i.e., Franck–Condon state) to the ICT state. Then the solvation and conformational relaxation processes took place from the ICT state to a ICT’ twisted solvent-stabilized state in polar solvents but not in non-polar one. This study also revealed that the π-conjugated linkers play an important role in modulating the ICT processes together with the medium polarity.

Dynamics of the ICT process in the excited states of a push–pull biphenyl derivative, 4-N,N-dimethylamino-4′-nitrobiphenyl (DNBP), has been investigated by Ghosh et al. [38]. Also in this case in high polarity solvents, e.g., acetonitrile, the ultrafast ICT process of DNBP was associated with the barrier twisting lowering of the N,N-dimethylaniline (DMA) group with respect to the nitrobenzene moiety giving rise to a TICT state. In solvents of moderate polarity, e.g., ethyl acetate, the twisting process rate was significantly slowed down with a consequent establishment of equilibrium between LE and TICT states due to the lowering of the energy barrier for their inter-conversion. Finally, in non-polar solvents, like cyclo-hexane, the authors observed a PICT in which the S_1_ state undergoes an ultrafast intersystem crossing to the triplet state because its energy lies very close to that of the T_2_ state.

The above-reported literature studies highlight the importance of the combination of ultrafast time-resolved spectroscopy and high quality computational methods. By this way it is possible to achieve a deep understanding of the ICT state and the chemical, physical and environmental parameters which are able to influence and tune it. This is crucial for the development of organic advanced optoelectronic materials. In this regard in a very recent paper Shen and coworkers reported on the strategies for stabilizing the ICT state [39]. As a matter of fact, ICT lifetime is very short, limiting the optoelectronic performance for some kind of applications. Usually short and well-conjugated bridges increase the rate of the charge separation process and facilitate charge migration, but the resulting stronger D–A interactions accelerate the charge recombination process shortening the ICT lifetime. Therefore, it is difficult to achieve fast charge transport and a long-lived ICT state at the same time. 

Shen and coworkers reported on the use of new foldamers of the protonated pyridine-modified tetraphenylethene derivatives that possess Through-Space Conjugation (TSC) characters as the models to study the impact of TSC on the ICT state [39]. TAS experiments illustrated that the lifetime of the ICT state in the molecule with strong TSC can be much longer than those of molecules without TSC, giving rise to a higher fluorescence quantum yield. By combining the theoretical calculations, based on DFT and TD-DFT, the authors demonstrated that the strong TSC can stabilize the ICT state and slow the charge recombination rate through an efficient dispersion of the charges. 

## 3. Recent Advances in Multidimensional Ultrafast Spectroscopy and Its Application in Unraveling the Intramolecular Charge Transfer (ICT) Nature of Push-Pull Molecular Systems

Multidimensionality is the natural development of the pump-probe spectroscopy. It has been implemented first in the Medium Infrared Region (MIR) [40,41] and more recently in the visible [42] and UV spectral regions [43]. Multidimensional optical spectroscopies have shown to be effective tools to understand the photophysics of a large set of extremely challenging phenomena such as quantum coherence in natural light harvesting, [42], structural dynamics [44], non-radiative relaxation, chemical reaction or solvation dynamics [45,46]. 

Advances in multidimensional ultrafast spectroscopies have been realized thanks to advances in femtosecond pulse generation, amplitude and phase control of laser light with pulse shapers such as Acousto-Optic Modulators (AOMs) or Spatial Light Modulators (SLMs) [47,48,49]. 

In the non-linear multidimensional spectroscopic techniques, the coherent higher order polarization of the sample is induced by a sequence of optical pulses and the parametric dependence of the signals on the time intervals between pulses is reach of pieces of information.

In a typical 2D spectroscopy experiment the sample is excited by three consecutive ultrashort pulses, with controllable relative delays. Interaction with pulse 1 generates a coherent superposition of oscillating dipoles, the interaction with pulse 2, delayed by a time *t*_1_ (coherence time), gives rise to a change in the population of the sample, finally, the interaction with the pulse 3, delayed by a time *t*_2_ (population time), leading to a macroscopic third-order nonlinear polarization *P*^(3)^(*t*_1_,*t*_2_,*t*_3_), which follows pulse 3 with a delay *t*_3_ and emits a nonlinear field *E*^(3)^(*t*_1_,*t*_2_,*t*_3_) ∝ *iP*^(3)^(*t*_1_,*t*_2_,*t*_3_). This field is collinearly superimposed with a pulse 4, i.e., the Local Oscillator (LO), and sent to a spectrometer, which performs a Fourier Transform (FT) with respect to *t*_3_, obtaining the detection frequency, i.e., *ω*_3_. Spectral interferometry between the nonlinear field and the LO allows full retrieval of the amplitude and phase of the nonlinear field *Ẽ*^(3)^(*t*_1_,*t*_2_,*ω*_3_), and thus of the nonlinear polarization $\tilde{P}$ ^(3)^(*t*_1_,*t*_2_,*ω*_3_). By performing another FT with respect to *t*_1_ for a fixed value of the waiting time *t*_2_, the 2D map $\tilde{P}$^(3)^(*ω*_1_,*t*_2_,*ω*_3_) is obtained, where *ω*_1_ is the excitation frequency [43].

Two main experimental configurations are used in 2D spectroscopy set-up, i.e., the non-collinear, heterodyne detected three-Pulse Photon Echo (3PPE) and the partially collinear Pump–Probe (PP) geometry as extensively reported in several works [43,49,50].

Vibrationally or electronically coupled molecules generate correlation plots, i.e., 2D maps, which reconstruct the dynamical events taking place during the controlled time intervals which can be interpreted in terms of multipoint correlation functions [51].

2D ultrafast spectroscopy allows the correlation between broadband excitation and emission frequencies as a function of system evolution, thus enabling the resolution of homogeneous (anti-diagonal) and inhomogeneous (diagonal) line shape components. Changes in the 2D line shapes give information on the Frequency–Frequency Correlation Function (FFCF) [51]. FFCF dynamics contain information about amplitudes and timescales associated with changes in the optical frequencies investigated which are related to molecular structure rearrangements and to solvation dynamics [47].

**Figure 4:**
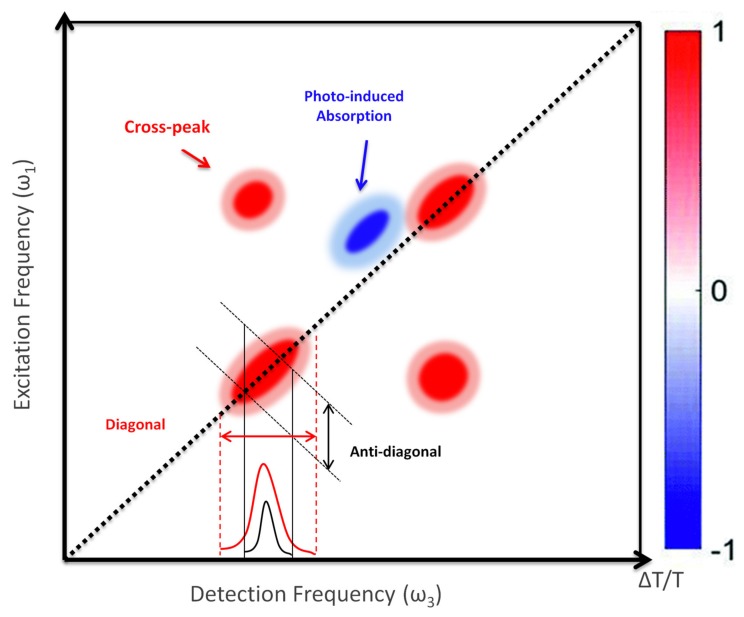
Illustration of a 2D map. The signals are characterized by diagonal peaks and cross peaks with both positive (GSB and SE) and negative Photo-Induced Absorption (P-IA) signals.

A typical 2D map is reported in Figure 4. It is characterized by positive differential transmission (*ΔT/T*) peaks along the diagonal, which correspond to the GSB and SE, of the transitions resonant with the excitation pulse. The map also contains positive peaks outside the diagonal, the so-called cross peaks, which identify the coupling between different transitions. A 2D map may also contain negative peaks due to Photo-Induced Absorption (P-IA), which correspond to transitions from bright or dark excited states or from the hot ground state.

Two-dimensional electronic spectroscopy (2DES) has been employed to investigate the electronic relaxation and energy transfer dynamics of molecules, molecular aggregates, and nanomaterials [45,52,53,54]. In these studies, it has been demonstrated the possibility of separating the homogenous and inhomogeneous broadening, identifying cross-peaks associated with energy transfer between excitons in biological systems or different electronic states of systems that undergoing fast non-radiative transitions. 

2DES has been recently employed together with high-level TD-DFT calculations to characterize ICT in two distyryl-functionalized Boron-dipyrromethene (BODIPY) compounds, i.e., 8-phenyl-3,5-di(diamino)styryl-borondipyrromethene (PHDB) and the analogous 8-(*p*-nitrophenyl)-3,5-di(diamino)styryl-borondipyrromethene (NO2-PHDB) in two different solvents THF and MeOH [55]. 2DES has shown to provide direct insight into the role of the solvent in the relaxation dynamics of the ICT in these push-pull molecules. The analysis of the 2D data in terms of 2D frequency–frequency decay associated maps provided the time constant of the relaxation process (strongly solvent dependent) but also the direct determination of the related reorganization energy. The sensitivity of the 2DES technique to vibrational coherence dynamics also allowed the identification of a possible relaxation mechanism involving specific interaction between vibrational modes of the dye and the solvent.

Also Lee and co-workers studied different BODIPY, i.e., BODIPY-2H and BODIPY-2I chromophores dissolved in methanol by applying 2DES [56]. The authors observed that after electronic excitation, the BODIPY molecules relax in response to the change in the charge distribution, through solvent reorganization and/or intra- and intermolecular vibrational energy redistribution. With the S_1_ state relaxation the energy gap decreases giving rise to a red shift of the SE signal. The authors observed ultrafast solvation dynamics of the two dyes on a sub-100 fs time scales. Furthermore, they demonstrated a new data analysis procedure for extracting the dynamic Stokes shift from 2DES spectra, focusing on monitoring the frequency changes associated with the SE, revealing an ultrafast solvent relaxation [56]. 

Multidimensional ultrafast spectroscopic techniques, and in particular 2DES, represent thus a powerful technique for the understanding of the ultrafast solvation dynamics of push-pull chromophores in various environments. 

## 4. Ab-Initio Approaches to Predict Charge Transfer Properties in Push-Pull Systems

As stated in Section 2, the delocalized π-system of push-pull molecules that span the electron donating and accepting groups in the outliers, confer them the property of an intrinsically charge-resonant framework between a neutral and zwitterionic form. It derives that their population can vary during photo-excitation and bespoke methods have been developed in the years to accurately predict the charge transfer character of these systems. In these regards, large improvements have been done in the ab-initio predictions of molecules and materials through DFT approaches [57]. TD-DFT calculations have been often pointed out to show discrepancies with experiments and those not depend on the chosen functional, but rather on the extent of electronic correlation [58,59]. Specific metrics were proposed in the years in order to quantifying the extent of charge transfer distance [60,61,62,63]. Descriptors based on electron density are considered universal [64], in particular the D_CT_ index relies on the barycenters of the densities associated with an electronic transition [65], while the φ_S_ index is based on the overlap between the attachment/detachment densities [66]. Furthermore, special purpose density-based indexes [67], have been recently proposed to disclose the extent of trustworthiness of charge-transfer description. Coulomb Attenuated Method in combination with the Becke’s three-parameter hybrid functional (B3) with the Lee, Yang, and Parr (LYP) expression for the nonlocal correlation (CAM-B3LYP) [68] and Becke’s Half-and-Half functional (BHandH) [69] were disclosed to provide the best agreement between computed and experimental vertical absorption energies for a set of push-and-pull systems based on 4,5-diyannoimidazole (DCI) as an acceptor group [12]. Range Separated Hybrid (RSH) functionals were proposed to predict with good accuracy the energetics of charge transfer states [70,71,72,73,74], achieving an agreement with experiments when combined with Polarized Continuum Models (PCM) [75]. Furthermore, the combination of Screened-Range Separated Hybrid (SRSH) functional with PCM [76,77,78] allows to predict CT states in condensed phases that accounts effectively for the electrostatic environment. In particular, the energetics of the weakly interacting D-A complexes of pentacene with C60 and poly-3-hexylthiophene (P3HT) calculated within the SRSH-PCM framework, was disclosed to well agree with the experimental energies in the condensed phase [77]. Another example of the SRSH-PCM scheme performance was reported for functionalized anthracenes as electron donors and tetracyanoethylene as acceptor both solvated in methylene chloride, where the calculated excited state energies accurately reproduced the measured benchmark values [79].

Surface hopping approaches were also shown to work very well to predict charge transfer rates and charge transport dynamics [80]. Spin-restricted ensemble-referenced Kohn-Sham (REKS) methods extended to low excited states were proposed to study π stacked ethylene or polyacene molecules, well predicting the double bond dissociation mechanisms [81]. 

In order to cut down the computational costs that are often required in ab-initio calculations, different methods were proposed. For instance, QM/MM scheme combined with an electrostatic embedding has been recently proposed to compute the PES surfaces of multichromophoric systems prior to surface hopping-based excited state dynamics [82]. 

Tight binding DFT (TBDFT) approaches have been proposed and were pointed out to work similarly to DFT methods but at smaller computational costs. TBDFT can be improved with fitting parameters on reliable exchange correlation functionals, an example is provided by the ones reconstructed through genetic algorithms [83]. Recent methods to calculate the first hyperpolarizability of extended conjugated systems include the simplified time-dependent density functional theory (sTD-DFT) working on approximation of the exchange integrals with short ranged damped Coulomb interactions of transition density monopoles or an implementation of sTD with TBDFT [84], as well as a combination of semi-empirical hamiltonians for speeding the calculations of excited state dynamics of solvated push-pull molecules [85]. 

### Selected Applications of Ab-Initio Predictions in Push-Pull Frameworks

Optical response is indeed subtly related to the typical photochemical pathways [86]. For instance, DFT simulations of donor–acceptor Stenhouse adducts have shown that the photocyclization switch highly weakens the first hyperpolarizability owing to the loss of conjugation that affects the non-linear optics responses [87]. Indeed, optical response is strongly affected from dihedral angle rotations [88,89,90]. The dependence of the dihedral angle on the extent of conjugation in push-pull systems was assessed through self-consistent charge density functional tight binding (SCC-DFTB) and compared with standard DFT functionals as B3LYP or PBE XC as well as MP2 methods [91]. TD-DFT simulations were useful to distinguish two different mechanisms occurring in molecular rotors with polar or non-polar solvents. Non-polar solvents were disclosed to induce fluorescence deactivation through the C=C twist rotation, whereas polar solvents hinder this twist, favoring a β twisted internal charge transfer state from which the non-radiative relaxation occurs [92]. A recent combination of non-adiabatic excited state dynamics coupled with molecular mechanics predicted the hindrance of trans-cis isomerization in push-pull azobenzene-based dye in the titanium dioxide (101) surface, owing to the ultrafast electron transfer to the titanium dioxide surface [93]. TD-DFT simulations based on hybrid B3LYP [69] or M06-2X [94] functionals and CAM–B3LYP [68] or ωB97X [95] long-range corrected functionals with the 6−31 + G*, aug–cc–pVDZn [96,97,98,99,100] predicted a sub-picosecond emitting state disentangled from a very close in energy fluorescence state related to the fully relaxed S1–S0 transition [101] of a cationic push-pull *o*-(*p*-dimethylamino-styryl)-methylpyridinium (DASPMI) dye.

The dependence of the photochemical properties on the physicochemical environment was assessed through Raman spectra and TD-DFT predictions [102]. Those highlighted the dependence of photodissociation processes occurring in push-pull molecules. In the case of 4-dimethylamino-4′-nitrostilbene no photo-reactions were proposed to occur in solution owing to the energy dissipation through the surrounding solvent molecules, whereas in solid state a thermal activation of the photo-reactions was rather proposed. In these regards, TD-DFT simulations pointed out the dependence in carbazole-based push-pull dyes of the lowest energy transition on the environment as solvent or temperature [103]. Indeed, non-equilibrium molecular dynamics simulations of liquid-deuterated methanol pointed out a fast non-thermal relaxation and thermal diffusion connected to the hydrogen-bond patterns and to the typical length-scale [104]. Both mechanisms concur in the observed signal of IR pump-probe spectroscopy, although thermal diffusion contribution was predicted to be reduced under short time-scales [104]. In these regards, simulations of two dimensional electronic spectra have been developed [105], together with a semi-classical path integral formalism [106]. Data from pump-probe spectroscopy were connected with molecular conformations, charge transfer and their related photo-responsive properties [107].

## 5. Outlooks on Machine Learning-Based Methods and Large-Scale Simulations of Light-Induced π-Delocalized Frameworks 

**Figure 5:**
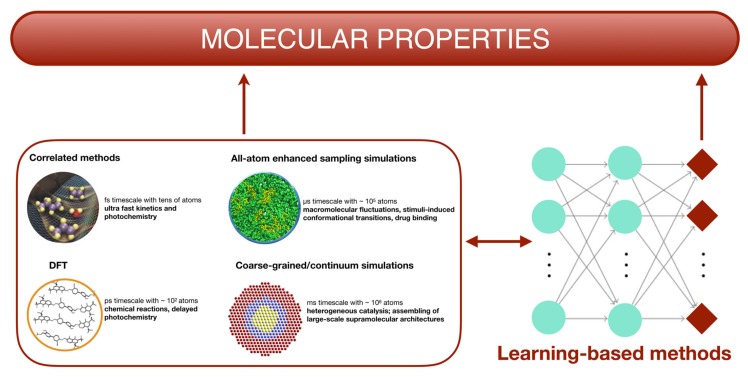
Timescale length governing the choice of the simulation technique used to predict a given chemical phenomenon. All the techniques can be in principle accelerated through learning methods, as machine or deep learning which includes neural networks, reported in the figure.

Practical ab-initio computations are limited to several hundreds of atoms with dynamics ranging in the order of picoseconds. This drawback pushed scientists to develop more accessible methods (Figure 5), keeping the accuracy of quantum chemical calculations.

In recent years, machine learning approaches have been proposed to accelerate calculations with reasonable accuracy [108,109], showing an interesting potential of transferability to larger molecular scaffolds [110,111] like representing the training sets through Hartree-Fock molecular orbitals (MOs) [111]. Indeed, solving the electronic state density thereby learning the density models through DFT-based datasets, instead of using the gradient descent that requires the calculation of the functional derivative, has been shown to break down the typical cost of DFT [112].

In these regards, deep learning based on multi-layers perceptrons was employed to predict average exciton transfer time for specific sets of protein-pigment complexes, as the Fenna-Matthews-Olson (FMO) complex, the reaction centers and core antennae CP43 or CP47 of photosystem II including the reaction centers [113] by training an artificial neural network based on initial sets of Frenkel exciton Hamiltonians. Spectral densities and exciton populations were also predicted for the first excited state of the FMO complex at a computational cost far less than half the time needed for TD-DFT with multi layers perceptrons trained on the excited state energies computed at QM/MM level, representing the molecular space within Coulomb matrices [114].

Indeed, following pioneering methods based on the representation of the QM potential energy surfaces through a neural network potential [115,116,117], a wealth of contributions has been rapidly increasing at an extraordinary pace. Accurate electron density models have been developed through machine learning approaches with the prospect to predict charge density of amino acids and their protein building blocks [118], as well as polarizability predictions through the combination of Linear Response Coupled Cluster Singles and Doubles (LR-CCSD) theory and symmetry-adapted machine-learning for various organic molecules [119]. The choice of pseudopotentials and basis sets in the training datasets has indeed a profound impact on the accuracy of the prediction towards the experimental value [120]. 

Machine learning potentials were developed for adiabatic states in the framework of fewest switches surface hopping for devising non-adiabatic excited state simulations [121], accelerating the timescale of the system. Deep neural networks have been also proposed to accelerate surface-hopping molecular dynamics for predicting photodissociation mechanisms [122] as well as path integral molecular dynamics coupled with machine learning-based force fields [123].

Machine learning techniques can be also coupled with enhanced sampling methods, as the smart protocol based on the expression of the bias potential as a neural network in the framework of enhanced sampling simulations [124] or a combination of variational Bayes methods within deep learning [125] as well as the construction of collective variables trough an artificial neural network [126].

For predicting conformational transitions occurring in large-sized systems, all-atom simulations combined with enhanced sampling are to date the computational technique that well balance accuracy and computational cost [127,128,129,130,131,132,133]. In this framework, excited state potentials have been developed in the years to study molecular conformations in a given electronic state in oligothiophene [134], as well as for predicting the absorption properties of the anthocyanidine dye in solution [135] or thermally activated delayed fluorescence emitters [136]. More recently, a scheme pioneered by some of us [137] introduced enhanced sampling simulations in the framework of well-tempered [138] metadynamics [139] with excited-state torsional potentials in combination with Free-Energy Perturbation (FEP) theory to estimate free-energy gaps from the ground to the excited states. The prospect of this simulation approach is to further proceed with the property prediction of large-scale molecular rotors and their optical response through light stimuli irradiation. 

## 6. Conclusions and Future Directions

In this review, we reported on the recent literature dealing with the characterization of the ICT state in push-pull molecules designed for various applications. In the first part we have demonstrated that TAS spectroscopy is as a very effective tool to understand the nature of the ICT and the influence of molecular structure, geometrical rearrangements and solvation dynamics on this transition. We have also reported about recent advances in ultrafast 2D spectroscopies focusing our attention on their potentiality in elucidating the role of the solvent in the relaxation dynamics of the ICT in push-pull molecules. In particular both TAS and 2DES highlight that polar solvents stabilize states with an increased dipole moment. Any excitation process implies a charge displacement. When this occurs on large molecular distances a geometrical rearrangement is required to make the ICT state more stable. Fluorescence lifetime is a consequence of an interconnection between different competitive relaxation phenomena. In this view if the relaxed structure is geometrically and solvent stabilized the competition between non-radiative and radiative processes favors the latter ones. 

Owing to the fact that a wealth of spectroscopic studies reported so far has been corroborated by DFT and TD-DFT, we discussed recent advances in the prediction of ground and excited state molecular properties through DFT methods. Those can predict with good accuracy structure and energetics, a feature that led to the popularity of DFT approaches, becoming straightforward and accessible at an extraordinary pace. We also discussed Machine learning based approaches together with free-energy simulation techniques combined with excited state potentials, recently proposed to predict the light-induced properties of large-scale systems.

The achievement of a thorough characterization of the ICT transition and the prediction of the photophysical and photochemical properties of push-pull systems can boost a specific design. In view of their peculiar light-response activation/deactivation pathways which depend on the specific excitation, we deem that a selection of push-pull molecules can be interesting photo-responsive systems with high tunable properties. Indeed, these molecules are very versatile, and we foresee a strong potential for photopharmacology applications, a growing and fertile research field that can be clinically relevant in the near future. A synergy between molecular simulations and ultrafast spectroscopy techniques can trigger innovative research frameworks enabling the realization of bespoke functional molecular scaffolds.
